# Fast atom transport and launching in a nonrigid trap

**DOI:** 10.1038/s41598-017-05823-x

**Published:** 2017-07-18

**Authors:** A. Tobalina, M. Palmero, S. Martínez-Garaot, J. G. Muga

**Affiliations:** Departamento de Química Física, UPV/EHU, B. Sarriena s/n, 48940 Leioa, Bizkaia Spain

## Abstract

We study the shuttling of an atom in a trap with controllable position and frequency. Using invariant-based inverse engineering, protocols in which the trap is simultaneously displaced and expanded are proposed to speed up transport between stationary trap locations as well as launching processes with narrow final-velocity distributions. Depending on the physical constraints imposed, either simultaneous or sequential approaches may be faster. We consider first a perfectly harmonic trap, and then extend the treatment to generic traps. Finally, we apply this general framework to a double-well potential to separate different motional states with different launching velocities.

## Introduction

**Figure 1:**
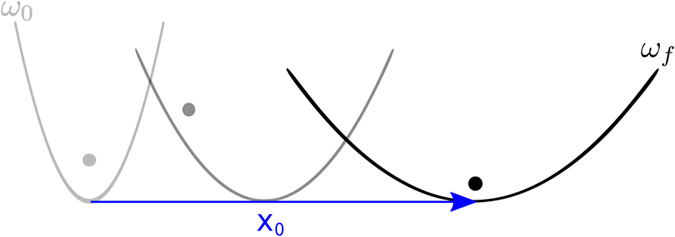
Scheme of the transport protocol with a change in the frequency of the trap.

An important goal of modern atomic physics is to control atomic motion for fundamental studies or to develop quantum-based technologies. Technological advances allow for driving individual atoms (ions^1, 2^ or neutral atoms^3^) along microscopic or mesoscopic predetermined space-time paths. This control will enable us to use the rich structure and interactions of ions and neutral atoms in circuits and devices where quantum phenomena play a significant role. Many operations require moving the atoms fast to keep quantum coherence, leaving them unexcited at their destination. Slow adiabatic shuttling may avoid excitation in principle, but the long times required make the processes prone to decoherence. Shortcuts to adiabaticity (STA)^4, 5^ are protocols for the control parameters that produce final states of an adiabatic process in much shorter times, typically via diabatic transitions at intermediate times. In this paper, we find STA to drive a single atom by a moving and nonrigid potential with time-dependent frequency as schematically shown in Fig. 1. We shall focus first on harmonic traps, and then a theory for more general potentials is also put forward. Two types of basic processes addressed are: (*i*) transport where the wave packet center and trap start and end at rest, and also (*ii*) launching or stopping processes, where the wave-packet center and trap start (resp. end) at rest, and ends (resp. start) with a nonzero velocity. Invariant-based inverse engineering has been applied to designing STA for rigid transport (with a constant potential in the moving frame)^6–8^, and trap expansions or compressions^4, 9–12^. While shuttling and expansion or compression could be performed sequentially, doing both operations simultaneously, as proposed here, may save time and offers broader control possibilities. “Dual-task” operations must thus be compared to sequential operations. In principle, STA for rigid transport and expansions can be done in arbitrarily short times, but only if infinite resources and energies are available, which is never the case in practice. Often, the control parameters cannot go beyond certain values. For example, a very fast trap expansion without final excitation needs transient imaginary frequencies of the external trap (a concave-down potential), which are not easy to implement in all trap types. In optical traps, for example, the passage through the atomic resonance of the laser frequency to go from a trap to an antitrap may produce undesired excitation. A different, common constraint is the limitation on the spatial domain allowed for the trap center. We shall show that, depending on the constraints imposed, either sequential or dual-task protocols may be faster.

There are different fields or applications where simultaneous transport and expansion or compression between initial and final states at rest is of relevance. In quantum heat engines and refrigerators^13–22^ for example, the (thermodynamically) adiabatic expansion or compression strokes of the cycle could be realized simultaneously transporting the quantum working medium between baths at different locations. Also, when expanding or separating ion chains, which are basic processes to develop a scalable quantum-information architecture^23^, the effective dynamics of the normal modes involves simultaneous transport and frequency change^24, 25^. One more scenario where transport and frequency change occur simultaneously is the bias inversion of an asymmetric double-well potential^26^.

Launching and stopping protocols are as well useful for many applications. An example of a stopping device is the “inverse coil gun” implemented by Mark Raizen and coworkers^27^. It uses pulsed magnetic fields to slow down a supersonic beam (e.g. from 500 to 50 m/s^27^) so as to leave the atoms ready for spectroscopic studies, controlled collisions, or further cooling techniques. One advantage of stopping techniques by magnetic (for paramagnetic species) or electric fields (for ions), is their broad range of applicability, beyond the very restricted class of atoms with a cycling transition that can be treated by standard laser cooling approaches. The opposite process, launching, is also of much current interest: launching ions with a specific speed is used in particular for their implantation or deposition^28^. Accurately controlled launching can contribute to different quantum technologies such as ion microscopy, those using a controlled “soft landing” of slow ions on a surface, and those controlling the location of defects (NV centers) that have been proposed for sensors and also as the basis of a possible architectures for quantum information processing. Deterministic sources of single cold ions have been proposed and demonstrated^28, 29^ that limit the position-momentum uncertainty only due to the Heisenberg principle. Our goal here is to control of the velocity, and its dispersion. This is facilitated by the possibility to change the trap frequency along the shuttling. Differential launching of different motional states is also possible as we shall demonstrate with a double well.

While the mathematical framework of this work is equally applicable to neutral atoms or trapped ions, the numerical examples make use of parameters adapted to trapped ions^1, 2^.

### Invariant-based inverse engineering

Lewis and Riesenfeld^30^ noted that the solutions of the Schrödinger equation for a time-dependent Hamiltonian can be written as superpositions of eigenstates of its dynamical invariants. Dhara and Lawande^31^ and Lewis and Leach^32^ worked out the details for a particle of mass *m* that evolves according to Hamiltonians of the form

1 $$H=\frac{{p}^{2}}{2m}-F(t)x+\frac{m}{2}{\omega }^{2}(t){x}^{2}+\frac{1}{{\rho }^{2}(t)}U[\frac{x-\alpha (t)}{\rho (t)}],$$

where *F*(*t*) is a homogeneous force, $\omega (t\mathrm{)/(2}\pi )$ the frequency of a harmonic term, *U* an arbitrary function, and *α*(*t*) and ρ(*t*) are auxiliary functions. *x* and *p* represent conjugate position and momentum operators of the particle.

The Hamiltonian in Eq. (1) has the quadratic-in-momentum invariant

2 $$I=\frac{1}{2m}{[\rho (p-m\dot{\alpha })-m\dot{\rho }(x-\alpha )]}^{2}+\frac{1}{2}m{\omega }_{0}^{2}{(\frac{x-\alpha }{\rho })}^{2}+U(\frac{x-\alpha }{\rho }),$$

where the dot means time derivative. *I* satisfies indeed the invariance equation

3 $$\frac{dI}{dt}\equiv \frac{\partial I(t)}{\partial t}+\frac{1}{i\hslash }[I(t),H(t)]=\mathrm{0,}$$

provided the scaling factor *ρ* and *α* satisfy the Ermakov and Newton equations,

4 $$\ddot{\rho }+{\omega }^{2}(t)\rho =\frac{{\omega }_{0}^{2}}{{\rho }^{3}},$$

5 $$\ddot{\alpha }+{\omega }^{2}(t)\alpha =\frac{F(t)}{m},$$

where *ω*_0_ is a constant. For simplicity we choose *ω*_0_ = *ω*(0).

Any wavefunction *ψ*(*t*) driven by the Hamiltonian (1) may be written in terms of eigenvectors *ψ*_*n*_ of the invariant (2),

6 $$\psi (x,t)=\sum _{n}{c}_{n}{e}^{i{\theta }_{n}}{\psi }_{n}(x,t),\quad I(t){\psi }_{n}(x,t)={\lambda }_{n}{\psi }_{n}(x,t),$$

where *c*_*n*_ are constant coefficients, the *λ*_*n*_ are the eigenvalues, and *θ*_*n*_ are Lewis-Riesenfeld phases that can be calculated from *H* and *ψ*_*n*_^30^, ${\theta }_{n}(t)=\frac{1}{\hslash }{\int }_{0}^{{t}_{f}}dt^{\prime} \langle {\psi }_{n}(t^{\prime} )|i\hslash \frac{\partial }{\partial t}-H(t^{\prime} )|{\psi }_{n}(t^{\prime} )\rangle \mathrm{.}$ The *ψ*_*n*_ have the form^31^

7 $${\psi }_{n}(x,t)={e}^{\frac{im}{\hslash }[\dot{\rho }{x}^{2}/2\rho +(\dot{\alpha }\rho -\alpha \dot{\rho })x/\rho ]}\frac{1}{{\rho }^{1/2}}{\varphi }_{n}(\frac{x-\alpha }{\rho }),$$

where the ${\varphi }_{n}(\sigma )$ (normalized in $\sigma :=\frac{x-\alpha }{\rho }$ space) are the solutions of the auxiliary, stationary Schrödinger equation

8 $$[-\frac{{\hslash }^{2}}{2m}\frac{{{\rm{\partial }}}^{2}}{{\rm{\partial }}{\sigma }^{2}}+\frac{1}{2}m{\omega }_{0}^{2}{\sigma }^{2}+U(\sigma )]{\varphi }_{n}(\sigma )={\lambda }_{n}{\varphi }_{n}(\sigma ).$$

The physical meaning of *α* is made evident in Eq. (7) as a centroid for the dynamical wavefunctions that satisfies the Newton equation (5). *α* is also the center of the potential term ${\rho }^{-2}U[(x-\alpha )/\rho ]$ when *U* does not vanish.

To inverse engineer the interaction between the initial time, *t* = 0, and a final time *t*_*f*_, we first set the initial and final Hamiltonians. For transport between stationary traps, commutativity is imposed between the Hamiltonian and the invariant at boundary times so that they share eigenstates. Thus the dynamics maps eigenstates of *H*(0) onto eigenstates of *H*(*t*_*f*_) via the corresponding invariant eigenstates, even though at intermediate times diabatic transitions may occur. The commutation of *H* and *I* at boundary times implies boundary conditions for *α*, *ρ*, and their derivatives. We design these functions to satisfy the necessary boundary conditions, and then, from the auxiliary Eqs (4) and (5) the control parameters $\omega (t)$ and $F(t)$ are found. For launching/stopping processes the invariant and Hamiltonian do not commute at final time in the laboratory frame, but the states may be chosen as eigenstates of the Hamiltonian in the comoving and coexpanding frame.

## Results

### Dual-task transport in a nonrigid harmonic trap

Let us assume first that the external trap is purely harmonic, i.e., we take *U* = 0 and $F=m{\omega }^{2}(t){x}_{0}(t)$, where ${x}_{0}(t)$ is the position of the trap center. Then, the Hamiltonian in Eq. (1) becomes, adding a purely time-dependent term that does not affect the physics to complete the square,

9 $$H=\frac{{p}^{2}}{2m}+\frac{1}{2}m{\omega }^{2}(t){[x-{x}_{0}(t)]}^{2}\mathrm{.}$$

The average energy for this system in the *n*th state (7) is given by

10 $$E=\langle H\rangle =\frac{(2n+1)\hslash }{4{\omega }_{0}}[{\dot{\rho }}^{2}+{\omega }^{2}(t){\rho }^{2}+\frac{{\omega }_{0}^{2}}{{\rho }^{2}}]+\frac{1}{2}m\dot{\alpha }+\frac{1}{2}m{\omega }^{2}(t){[\alpha -{x}_{0}(t)]}^{2}.$$

For rigid transport^6^, *ω* is constant and Eq. (4) is trivially satisfied for $\rho (t\mathrm{)=1}$. Here, the goal is to transport a particle a distance *d*, and additionally change the angular frequency of the trap from the initial value *ω*_0_ to the final value ${\omega }_{f}\equiv \omega ({t}_{f})={\omega }_{0}/{\gamma }^{2}$, without final excitation. The control parameters are the frequency ω(*t*) and the position of the center of the trap ${x}_{0}(t)$. Figure 1 shows schematically this process. The auxiliary functions *α*(*t*) and $\rho (t)$ have to satisfy the boundary conditions

11 $$\begin{array}{c}\alpha \mathrm{(0)}=\mathrm{0,}\quad \alpha ({t}_{f})=d,\\ \rho \mathrm{(0)}=\mathrm{1,}\quad \rho ({t}_{f})=\gamma \mathrm{.}\end{array}$$

We also find, by imposing commutativity between Hamiltonian and invariant at boundary times, the boundary conditions

12 $$\begin{array}{c}\dot{\alpha }\mathrm{(0)}=\dot{\alpha }({t}_{f})=\mathrm{0,}\\ \dot{\rho }\mathrm{(0)}=\dot{\rho }({t}_{f})=0.\end{array}$$

Additionally, to satisfy the invariant condition in Eq. (3) we need to impose

13 $$\begin{array}{c}\ddot{\alpha }\mathrm{(0)}=\ddot{\alpha }({t}_{f})=\mathrm{0,}\\ \ddot{\rho }\mathrm{(0)}=\ddot{\rho }({t}_{f})=0.\end{array}$$

Now, we may propose ansatzes that satisfy all boundary conditions in Eqs (11), (12) and (13). A simple choice is $\rho (t)={\sum }_{i\mathrm{=0}}^{5}{\rho }_{i}{s}^{i}$ and $\alpha (t)={\sum }_{i\mathrm{=0}}^{5}{\alpha }_{i}{s}^{i}$, where $s=t/{t}_{f}$. Fixing the coefficients ${\rho }_{i}$ and ${\alpha }_{i}$ to satisfy the boundary conditions, the auxiliary functions become

14 $$\rho (t)=1+\mathrm{10(}\gamma -\mathrm{1)}{s}^{3}-\mathrm{15(}\gamma -\mathrm{1)}{s}^{4}+\mathrm{6(}\gamma -\mathrm{1)}{s}^{5},\,\alpha (t)=10d{s}^{3}-15d{s}^{4}+6d{s}^{5}\mathrm{.}$$

Substituting $\rho$ in Eq. (4), the time dependent frequency in (9) takes the form

15 $$\omega (t)=\sqrt{\frac{{\omega }_{0}^{2}}{{\rho }^{4}}-\frac{\ddot{\rho }}{\rho }},$$

whereas, from Eq. (5), the transport function (position of the trap center) is

16 $${x}_{0}(t)=\frac{\ddot{\alpha }}{{\omega }^{2}}+\alpha ,$$

that can be now calculated with Eqs (14) and (15). The form of the polynomial for $\rho$ in Eq. (14) is not affected by the transport, so the function for the frequency in Eq. (15) is the same as the one used for pure expansions^4^. Similarly, the form of $\alpha (t)$ is not affected by the expansion, but the trap position ${x}_{0}(t)$ is different from the one in rigid transport^6^ due to the time dependence of the frequency. The dual task protocol is thus not just a simultaneous superposition of recipes for pure expansions and rigid transport but a genuinely different process.

We performed a number of tests to compare the times required by the sequential or dual protocols. In principle, both the sequential and the dual drivings can be done arbitrarily fast, if no limitations are imposed. However, subjected to technical limitations the minimal times may be different. One of the bounds will be to keep the frequency always real, ${\omega }^{2}(t) > 0$, since a repulsive parabola may be difficult to implement in some trapping methods. Other natural constraint is to limit the trap position bounded within the “box” [0, *d*].

We carry out the comparisons for a ^9^Be ^+^ion, shuttled over a distance $d=370$ *μ*m in a trap with initial frequency ${\omega }_{0}\mathrm{/(2}\pi )=2$ MHz expanded by a factor of 10, *γ* ^2^ = 10. For these parameters and polynomial ansatzes, the simple expansion has a minimal final time ${t}_{{f}_{exp}}^{(min)}=0.443$ *μ*s, below which imaginary frequencies appear. Note that this will also be the limit time before getting imaginary frequencies in the dual process, as Eq. (15) gives exactly the same evolution for *ω* in a simple expansion or a dual process. For rigid transport, carried out before the expansion at the highest trap frequency, the limit time is ${t}_{{f}_{tra}}^{(min)}=0.2$ *μ*s before exceeding the box. Thus, the total minimal time for the sequential protocol is ${t}_{{f}_{seq}}=0.643$ *μ*s. For the dual protocol, the minimal final time before exceeding the box is ${t}_{{f}_{dual}}=0.91$ *μ*s. Under the stated restrictions (real frequencies and the trap bounded by the predetermined box [0, *d*]), the dual protocol is slower than the sequential one, if performing the transport first and then the expansion. All final times are summarized in Table 1.

**Table 1:** Minimal times for the transport + expansion process when the trap frequency or/and center are limited, see text. Parameters: ${\rm{d}}=370$ *μ*m, $\gamma =\sqrt{10}$, and ${\omega }_{0}\mathrm{/(2}\pi )=2$ MHz.

	*ω* > 0	trap in [0, *d*]	Both conditions
Sequential	0.443 *μ*s	0.2 *μ*s	0.643 *μ*s
Dual	0.443 *μ*s	0.91 *μ*s	0.91 *μ*s

**Figure 2:**
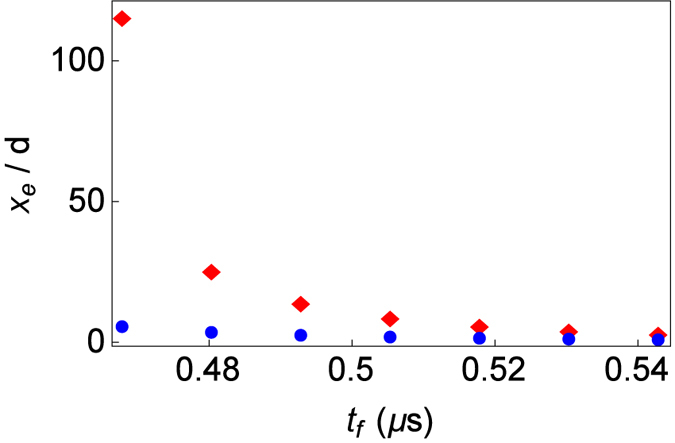
Ratio of the exceeded distance *x*_*e*_ and the transport distance *d* for the dual (blue circles) and sequential (red diamonds) non-rigid harmonic tranport protocols, for final times that do not require imaginary frequencies. Parameters used are $d=370$ *μ*m, $\gamma =\sqrt{10}$, and ${\omega }_{0}/\mathrm{(2}\pi )=2$ MHz.

If the only restriction is to keep real frequencies, dropping the limitation on the domain of the trap position, the minimal final time is in principle ${t}_{f}^{(min)}=0.443$ *μ*s for both the sequential and dual protocols, but in the sequential protocol this is a really challenging limit since the transport should be done in zero time. In both protocols the transport function exceeds the box [0, *d*]. In Fig. 2 we compare the ratio between the exceeded distance beyond [0, *d*] and *d* for the sequential and the dual drivings, with respect to the total process time. The exceeded distance is defined in terms of the maximum (${x}_{{0}_{max}}$) and the minimum (${x}_{{0}_{min}}$) values of the trajectory as ${x}_{e}={x}_{{0}_{max}}-{x}_{{0}_{min}}-d$. The figure shows that the dual protocol is much more robust. As the minimal possible time is approached, the ratio in the sequential protocol increases dramatically. In contrast, the ratio in the dual protocol is very stable, making potentially easier to perform the dual protocol for short times.

### Dual-task launching in a harmonic trap

We study now launching processes where the frequency of the trap is time dependent (stopping processes may be designed by inverting the launching protocols). If the ion is to be launched adiabatically with a very precise velocity, the trap should have a small final frequency to minimize the uncertainty. STA protocols will achieve the same goal in a shorter time.

The order of the sequence plays a relevant role to compare sequential or dual launching protocols. In the previous subsection, when the final state is at rest, the sequential protocol may be faster than the dual one when transport is done first, then the expansion. For the launching process, the only meaningful sequential process implies to expand first, and then to transport, but a small trap frequency does not enable us to implement a fast launching. It is therefore useful to combine the time dependences of frequency and displacement of the trap in a dual protocol.

The boundary conditions to be imposed for this launching protocol are the same as in Eqs (11), (12) and (13), except that the first derivative of *α* at final time, is now the final launching velocity ${v}_{f}$,

17 $$\dot{\alpha }({t}_{f})={v}_{f}\mathrm{.}$$

Additionally, boundary conditions are imposed on the third derivative of *α*,

18 $${\alpha }^{\mathrm{(3)}}\mathrm{(0)}={\alpha }^{\mathrm{(3)}}({t}_{f})=\mathrm{0,}$$

where ^(*n*)^ means $n$th derivative, so that, according to Eq. (16), the velocity of the trap ${\dot{x}}_{0}$ and the velocity of the wave packet $\dot{\alpha }$ are the same at the boundary times. In order to satisfy the additional boundary conditions, we consider a higher-order polynomial ansatz for $\alpha$, $\alpha ={\sum }_{i\mathrm{=0}}^{7}{\alpha }_{i}{s}^{i}$, which upon fixing parameters to satisfy all boundary conditions gives

19 $$\alpha (t)=\mathrm{5(7}d-3{t}_{f}{v}_{f}){s}^{4}-\mathrm{3(28}d-13{t}_{f}{v}_{f}){s}^{5}+\mathrm{2(35}d-17{t}_{f}{v}_{f}){s}^{6}-\mathrm{10(2}d-{t}_{f}{v}_{f}){s}^{7}\mathrm{.}$$

Boundary conditions for *ρ* are the same as in the previous subsection, so the same ansatz used in Eq. (14) is valid here. Thus, the evolution of the frequency is given in Eq. (15), while the evolution of the trap position is found substituting Eqs (15) and (19) into Eq. (16).

We evaluated the sequential and dual launching protocols limiting the frequencies to real values and the domain of the trap center to [0, *d*]. For the same parameters used in the previous subsection, and for a final velocity ${v}_{f}=10$ m/s, the minimal expansion time is the one given in the previous subsection, ${t}_{{f}_{exp}}^{(min)}=0.443$ *μ*s, as the expansion does not change for the new boundary conditions. The rigid transport, however, performed with the final trap frequency, can be done in a minimal time ${t}_{{f}_{tra}}^{(min)}\mathrm{=2.295}$ *μ*s without exceeding the box. Thus, the minimal sequential time is ${t}_{ftot}^{(min)}\mathrm{=2.734}$ *μ*s. For the dual protocol, the minimal time not exceeding the box is ${t}_{{f}_{dual}}=1.216$ $\mu$ s. The times are summarized in Table 2. Here the dual protocol clearly outperforms the sequential one.

**Table 2:** Minimal final times for the launching + expansion process with limited frequency or/and trap center, see text. Parameters: ${\rm{d}}=370$ *μ*m, $\gamma =\sqrt{10}$, ${v}_{f}=10$ m/s, and ${\omega }_{0}/\mathrm{(2}\pi )=2$ MHz.

	*ω* > 0	trap in [0, *d*]	Both conditions
Sequential	0.443 *μ*s	2.295 *μ*s	2.734 *μ*s
Dual	0.443 *μ*s	1.216 *μ*s	1.216 *μ*s

A control possibility we have for the dual process, which does not exist for the sequential one, is to design the launching with a given constant expanding velocity, i.e., we impose $\dot{\alpha }({t}_{f})={v}_{f}$ as before and also

20 $$\dot{\rho }({t}_{f})=\varepsilon \mathrm{.}$$

Additionally, boundary conditions may be imposed on the third derivative,

21 $${\rho }^{\mathrm{(3)}}\mathrm{(0)}={\rho }^{\mathrm{(3)}}({t}_{f})=\mathrm{0,}$$

so that, from Eq. (4), $\dot{\omega }\mathrm{(0)}=0$ and $\dot{\omega }({t}_{f})=-2\varepsilon {\omega }_{0}/{\gamma }^{3}$, which guarantees that the expansion velocity of the dynamical state matches that of the instantaneous eigenstates of the trap, consistently with the time derivative of $\rho ({t}_{f})=\sqrt{\frac{{\omega }_{0}}{{\omega }_{f}}}$.

For the polynomial ansatz $\rho ={\sum }_{i\mathrm{=0}}^{7}{\rho }_{i}{s}^{i}$ the coefficients are fixed to satisfy the boundary conditions,

22 $$\rho (t)=1+\mathrm{5(}-7+7\gamma -3\varepsilon {t}_{f}){s}^{4}-\mathrm{3(}-28+28\gamma -13\varepsilon {t}_{f}){s}^{5}+\mathrm{2(}-35+35\gamma -17\varepsilon {t}_{f}){s}^{6}-\mathrm{10(}-2+2\gamma -\varepsilon {t}_{f}){s}^{7}\mathrm{.}$$

With the evolutions considered in this section, either for the expanding or the nonexpanding launching, a state which is initially an eigenstate of *H*(0) will not become an eigenstate of the Hamiltonian $H({t}_{f})$. Instead, the state of the system at the end of the process is, see Eq. (7), ${\psi }_{n}(x,{t}_{f})={e}^{\frac{im}{\hslash }[\varepsilon {x}^{2}/2\gamma +({v}_{f}\gamma -d\varepsilon )x/\gamma ]}\frac{1}{{\gamma }^{1/2}}{\phi }_{n}(\frac{x-d}{\gamma }),$ which can be shown to correspond to the Hamiltonian eigenstate in the moving and expanding reference system of the trap (see Methods).

The expectation value of the velocity for ${\psi }_{n}(x,{t}_{f})$ is ${v}_{f}$ and its dispersion is

23 $${\rm{\Delta }}v=\sqrt{\frac{\hslash (2n+1)}{2m{\omega }_{0}}({\gamma }^{2}{\varepsilon }^{2}+\frac{{\omega }_{0}^{2}}{{\gamma }^{4}})},$$

minimal with respect to $\varepsilon$ for $\varepsilon =0$. It can be lowered further by decreasing the final trap frequency (increasing $\gamma$). This result may be compared with the process where the initial trap is turned off and a constant electric field is applied. Then the dispersion does not change, ${\rm{\Delta }}v=\sqrt{[\hslash \mathrm{(2}n+\mathrm{1)}{\omega }_{0}\mathrm{]/(2}m)}\mathrm{.}$ Much smaller spreads can be achieved by the dual protocol, but $\gamma$ cannot be made arbitrarily small in a fixed process time. In particular, the requirement of keeping the frequency real implies the bound^9, 14^ ${t}_{f} > \sqrt{{\gamma }^{2}-1}/{\omega }_{0}$. A constant electric field has its own, different limitations, in particular, with constant acceleration the time is fixed as ${t}_{f}\mathrm{=2}d/{v}_{f}$ to reach a given final velocity ${v}_{f}$ in a distance $d$.

### Dual-task shortcuts in an arbitrary trap

Now, we extend the analysis to move and expand or compress an arbitrary confining potential from $U(x)$ to $\frac{1}{\rho {({t}_{f})}^{2}}U[\frac{x-\alpha ({t}_{f})}{\rho ({t}_{f})}]$. To stay within the family of processes described by Eq. (1), so that invariants are known, we must impose that the harmonic and linear terms depending on ${\omega }^{2}$ and $F$ vanish at the boundary times. We thus set ${\omega }_{0}=0$ hereafter. If initial and final potentials are at rest, by imposing commutativity between the Hamiltonian (1) and the invariant (2) and continuity at the boundary times, we get the same boundary conditions as in Eqs (12) and (13). We must also impose the boundary conditions in Eq. (11) for the system to be displaced and expanded or compressed, noting that now the constant $\gamma$ is not related to ${\omega }_{0}$. With these boundary conditions, using the auxiliary Eqs (4) and (5), $F\mathrm{(0)}=F({t}_{f})=\omega \mathrm{(0)}=\omega ({t}_{f})=0$. That is, the only non vanishing term of the potential at the boundary times ${t}_{b}\mathrm{=0,}\,{t}_{f}$ is $V({t}_{b})=\frac{1}{\rho {({t}_{b})}^{2}}U[\frac{x-\alpha ({t}_{b})}{\rho ({t}_{b})}].$ We design the functions $\alpha (t)$ and $\rho (t)$ polynomially as before, so that they satisfy all boundary conditions, and introduce them in the auxiliary equations to inversely obtain the control parameters. The auxiliary functions can be the same as in Eq. (14). Substituting $\rho$ in Eq. (4),

24 $${\omega }^{2}(t)=-\frac{\ddot{\rho }}{\rho },$$

and substituting this result and $\alpha$ in Eq. (5) we get

25 $$F(t)=m\ddot{\alpha }+m{\omega }^{2}\alpha \mathrm{.}$$

In other words, the protocol requires auxiliary time-dependent linear and quadratic potential terms apart from the scaled potential $\frac{1}{{\rho }^{2}(t)}U[\frac{x-\alpha (t)}{\rho }]$. This protocol is of course technically more demanding than the one designed for the simple harmonic trap, because of the need to implement and control all terms (linear, quadratic, and *U*-term) of the Hamiltonian (1).

The results can be extended to a launching scenario. To be specific, we shall consider the double well, a paradigmatic quantum model that has been used, for example, to study and control some of the most fundamental quantum effects, like interference or tunneling. With the advent of ultracold-atom-based technology, it also finds applications in metrology, sensors, and the implementation of basic operations for quantum information processing, like separation or recombination of ions^24^, as well as Fock state creation^33^, and multiplexing/demultiplexing vibrational modes^34, 35^. Here, we explore the possibility of using it for differential launching of vibrational modes.

We set *U* (in $\sigma :=\frac{x-\alpha }{\rho }$ space) as

26 $$U(\sigma )=\beta {\sigma }^{4}+\lambda {\sigma }^{2}+\mu \sigma ,$$

where *β*, *λ* and *μ* are constant parameters. *β*, is positive and *λ* negative so that we have indeed a double well. The linear term produces a bias between the wells. The condition^26^ $|\mu |\ll \frac{4\sqrt{2}}{3}\sqrt{-\frac{{\lambda }^{3}}{\beta }}$ enables us to approximate $U(\sigma )$ as the sum of two harmonic potentials with minima at^26^

27 $${\sigma }_{\pm }(t)=\pm \frac{1}{\sqrt{2}}\sqrt{-\frac{\lambda }{\beta }}+\frac{\mu }{4\lambda }$$

in *σ*-space, and effective angular frequency

28 $${\rm{\Omega }}=2\sqrt{-\frac{\lambda }{m}}\mathrm{.}$$

Limiting the linear coefficient as $|\mu | < \hslash {\mathrm{(2}\beta /m)}^{\mathrm{1/2}}$, the first excited and ground states lie in different wells^34^. We want to implement a protocol with a nonzero final expansion velocity, such that the effective launching velocities for ground and first excited states are different so that they separate further. We choose the boundary conditions for the auxiliary functions in Eqs (11) and (13) and for the first derivatives

29 $$\begin{array}{c}\dot{\alpha }\mathrm{(0)}=\mathrm{0,}\,\dot{\alpha }({t}_{f})={v}_{0},\\ \dot{\rho }\mathrm{(0)}=\mathrm{0,}\,\dot{\rho }({t}_{f})=\varepsilon \mathrm{.}\end{array}$$

Here the boundary conditions for the third derivatives [Eqs (18) and (21)] are not necessary. With these conditions, using fifth-order polynomial ansatzes, the auxiliary functions are finally given by

30 $$\alpha (t)=\mathrm{2(5}d-2{t}_{f}{v}_{0}){s}^{3}+(-15d+7{t}_{f}{v}_{0}){s}^{4}+\mathrm{3(2}d-{t}_{f}{v}_{0}){s}^{5},$$

31 $$\rho (t\mathrm{)=1}+\mathrm{2(}-5+5\gamma -2{t}_{f}\varepsilon ){s}^{3}+\mathrm{(15}-15\gamma +7{t}_{f}\varepsilon ){s}^{4}+\mathrm{3(}-2+2\gamma -{t}_{f}\varepsilon ){s}^{5}\mathrm{.}$$

**Figure 3:**
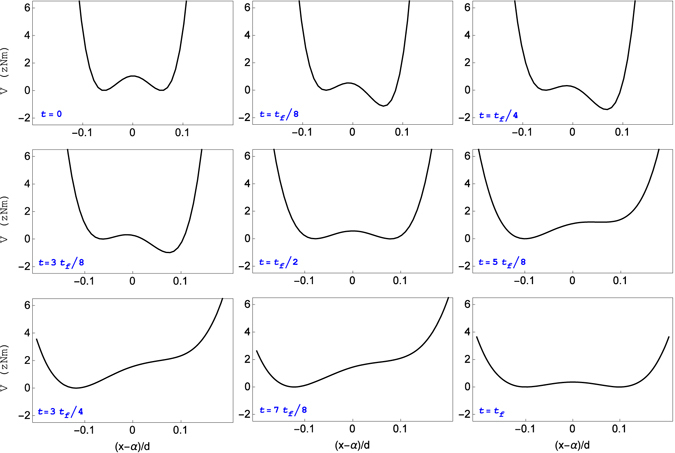
Time evolution of the shape of the launching double-well potential with velocities ${v}_{f}=10$ m/s and $\varepsilon \mathrm{=2/}s$. Each snapshot has been vertically displaced, without affecting the dynamics of the system, so that the minimum of the left well always lies at zero potential. The parameters used are $\lambda =-4.7$ pN/m, $\beta =5.2$ mN/m^3^, $\mu =86.4$ zN, $d=370$ *μ*m, $\gamma =\sqrt{3}$ and ${t}_{f}=1$ *μ*s. Even though not appreciated by the naked eye in the scale of the figures, the initial and final left wells are deeper than the right wells.

**Figure 4:**
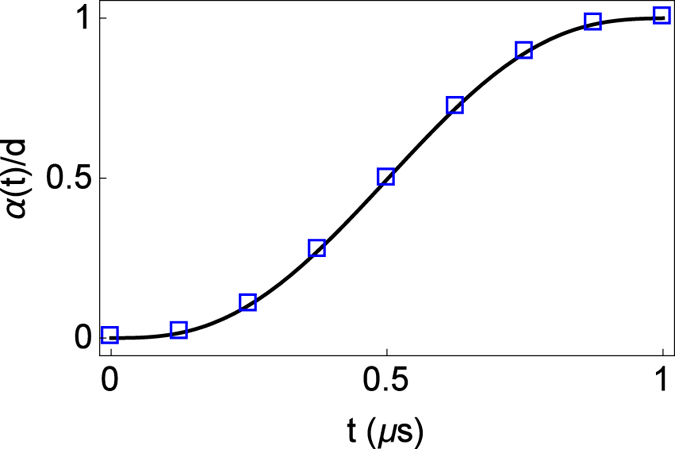
Scaled trajectory *α*, Eq. (30), of the center of the trap in a double well launching protocol with parameters $\lambda =-4.7$ pN/m, $\beta \,=\,5.2$ mN/m^3^, $\mu =86.4$ zN, ${\rm{d}}=370$ *μ*m, $\gamma =\sqrt{3}$ and ${t}_{f}\mathrm{=1}$ *μ*s, and velocities ${v}_{f}=10$ m/s and $\varepsilon =\mathrm{2/}s$. Blue rectangles mark the points of the trajectory in which a snapshot of the potential is depicted in Fig. 3.

These parameters directly give us the evolution of the potential term ${\rho }^{-2}U[(x-\alpha )/\rho ]$. The auxiliary harmonic and linear terms in the total Hamiltonian (1) are found by substituting $\alpha$ and $\rho$ in Eqs (24) and (25), respectively. The resulting potential (the sum of the three potential terms in Eq. (1)) is depicted in Fig. 3 as a function of $(x-\alpha )/d$, with $\alpha$ depicted in Fig. 4.

For this evolution, we can calculate the average final velocity of the ground states in each well, and the final dispersion,

32 $$\langle {v}_{\pm }\rangle ={v}_{0}+\varepsilon (\frac{\mu }{4\lambda }\pm \frac{1}{\sqrt{2}}\sqrt{-\frac{\lambda }{\beta }}),\,\,\,\,\,\,{\rm{\Delta }}v=\sqrt{\frac{\hslash }{4\sqrt{-m\lambda }}({\varepsilon }^{2}-\frac{4\lambda }{m{\gamma }^{2}})},$$

which is the same in both wells, as the effective frequency is also equal. Details of these calculations are displayed in Methods. Choosing the parameters so that $\langle {v}_{+}\rangle -\langle {v}_{-}\rangle \, > \,2{\rm{\Delta }}v,$ guarantees that the wave packets of each well will never overlap.

## Discussion

In this paper, we have used the invariant-based inverse-engineering method to design shortcuts to adiabaticity for nonrigid driven transport and launching. Shortcuts for a harmonic trap are designed first, and then the analysis is extended to an arbitrary trapping potential. Compared to rigid transport^6^, nonrigid transport requieres a more demanding manipulation, but it also provides a wider range of control opportunities, for example to achieve narrow final velocity distributions in a launching process, suitable for accurate ion implantation or low-energy scattering experiments. A further example is the possibility to launch the ground states of each well in a double well with different velocities. In a previous work^34^ processes to separate the ground and the first-excited states of a harmonic trap into different wells of a biased double well using STA were described. The processes discussed here can be applied to different systems such as neutral atoms in optical traps, or classical mechanical oscillators, for which, mutatis mutandis, most of the results apply.

## Methods

### Unitary diplacement and dilatation transformations

First, we prove that given an arbitrary unitary transformation *U*, the transformed invariant $I^{\prime} =UI{U}^{\dagger }$ is an invariant of the effective Hamiltonian $H^{\prime} =UH{U}^{\dagger }+i\hslash \frac{\partial U}{\partial t}{U}^{\dagger }$. Their commutator is given by

33 $$[I^{\prime} ,H^{\prime} ]=[UH{U}^{\dagger }+i\hslash \frac{\partial U}{\partial t}{U}^{\dagger },UI{U}^{\dagger }]=U[H,I]{U}^{\dagger }+i\hslash \frac{\partial U}{\partial t}I{U}^{\dagger }-i\hslash UI{U}^{\dagger }\frac{\partial U}{\partial t}{U}^{\dagger },$$

and the invariance condition [see Eq. (3)] for the transformed operators is satisfied,

34 $$i\hslash \frac{{\rm{\partial }}{I}^{{\rm{^{\prime} }}}}{{\rm{\partial }}t}-[{I}^{{\rm{^{\prime} }}},{H}^{{\rm{^{\prime} }}}]=U(i\hslash \frac{{\rm{\partial }}I}{{\rm{\partial }}t}-[H,I]){U}^{\dagger }=0.$$

Now we introduce the specific unitary time-dependent operator $U={U}_{{d}_{2}}\,{U}_{{d}_{1}}\,{U}_{p}\,{U}_{x}$. Operators ${U}_{{d}_{1}}$ and ${U}_{{d}_{2}}$ perform a time-dependent dilatation, and ${U}_{x}$ and ${U}_{p}$ a time-dependent translation in space and momentum, and are given by^36^

35 $$\begin{array}{c}{U}_{{d}_{1}}={e}^{-\frac{im\dot{\rho }}{2\hslash \rho }{x}^{2}};\quad {U}_{{d}_{2}}={e}^{\frac{iln\rho }{2\hslash }(px+xp)};\\ {U}_{p}={e}^{-\frac{im\dot{\alpha }}{\hslash }x};\quad {U}_{x}={e}^{\frac{i\alpha }{\hslash }x}\mathrm{.}\end{array}$$

In the comoving and coexpanding frame defined by this transformation, the new invariant

36 $$I^{\prime} =UI{U}^{\dagger }={U}_{{d}_{2}}\,{U}_{{d}_{1}}\,{U}_{p}\,{U}_{x}\,I\,{U}_{x}^{\dagger }\,{U}_{p}^{\dagger }\,{U}_{{d}_{1}}^{\dagger }\,{U}_{{d}_{2}}^{\dagger }=\frac{1}{2m}{p}^{2}+\frac{1}{2}m{\omega }_{0}{x}^{2}+U(x),$$

becomes time independent^37^. Note that *I*′ has the same form of the Hamiltonian in Eq. (8) and therefore, the eigenstates of *I*′ are given by ${\varphi }_{n}(x)$. The inverse transformation acting on ${\varphi }_{n}$ provides the time dependent eigenvectors of *I*(*t*) in Eq. (7),

37 $${\psi }_{n}(x,t)={U}^{\dagger }{\varphi }_{n}(x)={U}_{x}^{\dagger }\,{U}_{p}^{\dagger }\,{U}_{{d}_{1}}^{\dagger }\,{U}_{{d}_{2}}^{\dagger }\,{\varphi }_{n}^{{}^{^{\prime} }}(x)={e}^{\frac{im}{\hslash }[\dot{\rho }{x}^{2}/2\rho +(\dot{\alpha }\rho -\alpha \dot{\rho })x/\rho ]}\frac{1}{\sqrt{\rho }}{\varphi }_{n}(\frac{x-\alpha }{\rho }).$$

The Hamiltonian in the comoving and coexpanding frame is

38 $${H}^{{\rm{^{\prime} }}}=UH{U}^{\dagger }+i\hslash \frac{{\rm{\partial }}U}{{\rm{\partial }}t}{U}^{\dagger }=\frac{1}{{\rho }^{2}}(\frac{1}{2m}{p}^{2}+\frac{1}{2}m{\omega }_{0}{x}^{2}+U(x))+\frac{m}{2}(\frac{\ddot{\rho }{\alpha }^{2}}{\rho }-{\dot{\alpha }}^{2})-m\ddot{\alpha }\alpha ,$$

which, up to global terms that depend only on time, is proportional to the transformed invariant (36), so they commute at all times and thereby, share eigenstates at all times.

Note that the noninertial frame considered is comoving with *α*, which is the center of the term ${\rho }^{-2}U[(x-\alpha )/\rho ]$, but not necessarily the center of the harmonic potential $\frac{m}{2}{\omega }^{2}{(x-{x}_{0})}^{2}$ in Eq. (9) when *U* = 0. However, the boundary conditions are set, see Eq. (18), so that indeed the frames moving with *α* and ${x}_{0}$ coincide at boundary times ${t}_{b}\,=\,\mathrm{0,}\,{t}_{f}$, as $\alpha ({t}_{b})={x}_{0}({t}_{b})$, and $\dot{\alpha }({t}_{b})={\dot{x}}_{0}({t}_{b})$. Similarly Eq. (21) implies that the coexpanding frame depending on $\rho$ agrees with the one defined by the scaling factor ${\rho }_{trap}=\sqrt{{\omega }_{0}/{\omega }_{f}}$ associated with the expansion of the trap, $\rho ({t}_{b})={\rho }_{trap}({t}_{b})$, and $\dot{\rho }({t}_{b})={\dot{\rho }}_{trap}({t}_{b})$.

### Average velocity and dispersion in a double well

Here, we consider the Hamiltonian in Eq. (1) with $U(\sigma )$ given by a double well, Eq. (26), where ground and first-excited states lie in different wells and may be approximated by ground states of corresponding harmonic oscillators centered in ${\sigma }_{\pm }$ [see Eq. (27)], and effective angular frequency ${\rm{\Omega }}$ [see Eq. (28)]. If the initial state is either the ground or first-excited state, the dynamical state of the system is in either case

39 $${\psi }^{\pm }(x,t)={e}^{\frac{im}{\hslash }[\dot{\rho }{x}^{2}\mathrm{/2}\rho +(\dot{\alpha }\rho -\alpha \dot{\rho })x/\rho ]}\,\frac{1}{{\rho }^{\mathrm{1/2}}}\,{\phi }_{0}^{\pm },$$

where $${\varphi }_{0}^{\pm }={(\frac{m{\rm{\Omega }}}{\pi \hslash })}^{1/4}{e}^{-\frac{m{\rm{\Omega }}}{2\hslash }{(\frac{x-\alpha }{\rho }-{\sigma }_{\pm })}^{2}}{H}_{0}[\sqrt{\frac{m{\rm{\Omega }}}{\hslash }}\quad (\frac{x-\alpha }{\rho }-{\sigma }_{\pm })].$$

Using standard properties of Hermite polynomials the average of the velocity and its square are found to be

40 $$\langle {v}_{\pm }\rangle =-\frac{i\hslash }{m}\int {({\psi }^{\pm })}^{\ast }{\partial }_{x}{\psi }^{\pm }\,dx=\dot{\alpha }+\dot{\rho }\,{\sigma }_{\pm },$$

41 $$\langle {v}_{\pm }^{2}\rangle =-\frac{{\hslash }^{2}}{{m}^{2}}\int {({\psi }^{\pm })}^{\ast }{{\rm{\partial }}}_{x}^{2}{\psi }^{\pm }\,dx=(\dot{\alpha }+\dot{\rho }{\sigma }_{\pm }{)}^{2}+\frac{\hslash }{2m{\rm{\Omega }}}({\dot{\rho }}^{2}+\frac{{{\rm{\Omega }}}^{2}}{{\rho }^{2}}).$$

Finally, the dispersion, common to both wells, is given by

42 $${\rm{\Delta }}v={\rm{\Delta }}{v}_{\pm }=\sqrt{\langle {v}_{\pm }^{2}\rangle -{\langle {v}_{\pm }\rangle }^{2}}=\sqrt{\frac{\hslash }{2m{\rm{\Omega }}}({\dot{\rho }}^{2}+\frac{{{\rm{\Omega }}}^{2}}{{\rho }^{2}})}.$$

Equation (32) follows by substituting in Eqs (40) and (42) the expressions for ${\sigma }_{\pm }$ and Ω, Eqs (27) and (28), and the final values of the auxilary functions and their derivatives in Eqs (11) and (29).
